# 14-year hip survivorship after periacetabular osteotomy: a follow-up study on 1,385 hips

**DOI:** 10.1080/17453674.2020.1731159

**Published:** 2020-02-28

**Authors:** Josefine Beck Larsen, Inger Mechlenburg, Stig Storgaard Jakobsen, Theis Munchholm Thilleman, Kjeld Søballe

**Affiliations:** Department of Orthopaedic Surgery, Aarhus University Hospital, Aarhus N, Denmark

## Abstract

Background and purpose — Few studies have evaluated the long- and mid-term outcomes after minimally invasive periacetabular osteotomy (PAO). We investigated: (1) the long-term hip survival rate after PAO; (2) the risk of complications and additional surgery after PAO; and (3) the hip function at different follow-up points.

Patients and methods — We reviewed 1,385 hips (1,126 patients) who underwent PAO between January 2004 and December 2017. Through inquiry to the Danish National Patient Registry we identified conversions to total hip arthroplasty (THA) and complications after PAO. We evaluated the Hip disability and Osteoarthritis Outcome Score (HOOS) obtained preoperatively, and at 6 months, 2-, 5-, and 10-years’ follow-up.

Results — 73 of the 1,385 hips were converted to THA. The overall Kaplan–Meier hip survival rate was 80% (95% CI 68–88) at 14 years with a mean follow-up of 5 years (0.03–14). 1.1% of the hips had a complication requiring surgical intervention. The most common additional surgery was removal of screws (13%) and 11% received a hip arthroscopy. At the 2-year follow-up, HOOS pain improved by a mean of 26 points (CI 24–28) and a HOOS pain score > 50 was observed in 86%.

Interpretation — PAO preserved 4 of 5 hips at 14 years, with higher age leading to lower survivorship. The PAO technique was shown to be safe; 1.1% of patients had a complication that demanded surgical intervention. The majority of the patients with preserved hips have no or low pain. The operation is effective with a good clinical outcome.

Periacetabular osteotomy (PAO) is the most common surgical procedure to treat symptomatic hip dysplasia (Ganz et al. 1988, Clohisy et al. 2009). Previous studies have reported a 10-year hip survivorship of 78–95% in patients undergoing PAO. These studies, however, only include a small number of hips and surgical procedures performed during the surgical learning curve (Steppacher et al. 2008, Matheney et al. 2009, Hartig-Andreasen et al. 2012, Albers et al. 2013, Lerch et al. 2017, Ziran et al. 2018).

In addition to hip survivorship, several studies have investigated the risk of complications following PAO. It has been estimated that early serious complications occurred in 6–37% of patients (Clohisy et al. 2009). Delayed complication rates suggested that 9% of patients had major complications requiring surgical or arthroscopic intervention, including nonunion, hematoma/deep infection, revision PAO, heterotopic ossification, intraoperative fractures, osteotomy, or sciatic nerve damage (Wells et al. 2018b). To our knowledge, only a few studies have evaluated the long-term complications after PAO (Wells et al. 2018b).

Moreover, conversion to total hip arthroplasty (THA) may not be sufficient to describe the outcome after PAO, since patients may not want a THA or surgeons may not recommend it. Patient-reported outcomes (PRO) can therefore supplement the evaluation of the outcome after PAO. Previous studies have used different PROs to identify a failure after PAO, including the Merle d’Aubigné–Postel score < 15 or the Western Ontario and McMaster Universities Osteoarthritis Index (WOMAC) ≥ 10 (Matheney et al. 2009, Hartig-Andreasen et al. 2012, Albers et al. 2013, Lerch et al. 2017, Wells et al. 2018a). In this study, we used the Hip disability and Osteoarthritis Outcome Score (HOOS).

This study determines (1) long-term hip survival rate after PAO; (2) risk of complications after PAO; (3) hip function using HOOS at different follow-up points.

## Patients and methods

### Institutional database

Demographic, clinical, and PROs were collected in an institutional database that contains data from patients undergoing PAO at Aarhus University Hospital, Denmark and Mølholm Private Hospital, Denmark from 1998. The database was created in 2010 and includes prospectively gathered data from that time. Data on patients operated from 2004 to 2010 were retrospectively entered into the database in 2014.

Data on all patients included: sex, age at surgery, and right or left hip on which PAO was performed. Since 2010, preoperative demographic data have included: height, weight, BMI, educational level, pain measured on a visual analogue scale (VAS) during rest and during activity, center–edge (CE) angle, acetabular index (AI) angle, degree of osteoarthritis (OA), impingement test score, and previous treatment in the same hip. PRO was systematically gathered prospectively from 2010 and included HOOS obtained preoperatively and at 6 months, 2, 5, and 10 years postoperatively. For patients operated in 2004 and onwards, HOOS was available at 10 years postoperatively. For patients operated in 2009 and onwards, HOOS was available at 5 years postoperatively.

#### The Danish National Patient Registry

The Danish National Patient Registry (DNPR) is a national registry established in 1976 containing information on all contacts, surgical procedures, and admissions for patients treated at Danish hospitals. The DNPR contains information on dates of admission, and discharge diagnoses according to the International Classification of Diseases (ICD 10). Furthermore, the registry holds data on dates and types of surgical procedures, according to the Health Care Classification System, e.g. SKS codes for the hip (KNF) and pelvis (KNE) (Sundhedsdatastyrelsen 2019, see also Schmidt et al. 2014).

The patient data from our institutional database were merged with data from the DNPR regarding information on (1) THA, and (2) complications including deep vein thrombosis (DVT) and pulmonary embolism 1 month after PAO and information on which surgical procedures had been performed on the pelvis and hip. Furthermore, information on death and emigration was collected, where the DNPR allows for almost complete follow-up (Schmidt et al. 2014). Because most operations require a visit to the hospital and hospital encounters are registered consistently in the DNPR, a high level of completeness is expected (Schmidt et al. 2015). Since the DNPR contains all this information it allows for complete follow-up on all patients.

### Study population

**Figure 1.**
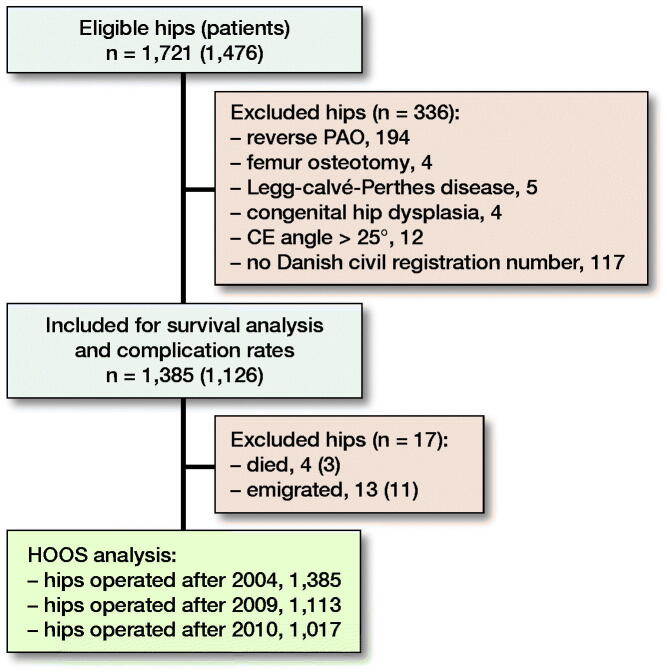
Flowchart showing the study population, reasons for exclusion, and number of patients included for survival analysis, complication rates, and Hip disability and Osteoarthritis Outcome Score (HOOS) analysis. PAO = periacetabular osteotomy. CE angle = center–edge.

Patients were identified from our institutional database on patients undergoing PAO. 1,721 surgically treated hips between January 2004 and December 2017 were eligible for inclusion in this study (Figure 1). The vast majority of operations were performed by the senior author (KS); the rest were performed (parts of or entirely) by 4 surgical fellows, thus minimizing the surgical learning curve. Concomitant hip arthroscopy is not performed at our institution. The exclusion criteria were reverse PAO, femur osteotomy, persons without a Danish civil registration number (patients from abroad), Legg–Calvé–Perthes disease, and congenital hip dislocation. 1,385 hips (1,126 patients) met the inclusion criteria. During the study 3 patients died unrelated to the operation (4 hips) and 11 patients emigrated (13 hips).

Indications for PAO surgery throughout the study were (1) symptomatic dysplasia of the hip with persistent hip pain and reduced function, (2) CE angle according to Wiberg < 25°, (3) pelvic bone maturity, (4) absence of hip subluxation, (5) internal rotation > 15°, and (6) hip flexion > 110°. Contraindications for PAO were (1) OA (this contraindication has gradually changed to exclude any OA above Tönnis Grade 1 [Tönnis 1987] from PAO surgery), (2) reduced ROM indicating joint degeneration, (3) lack of hip congruence and (4) BMI > 30. From 2016 the inclusion criteria for surgery changed for (1) OA = 0 (Tönnis 1987) (4) BMI ≤ 25 and age ≤ 45 years. The PAOs were performed using the minimally invasive transartorial approach developed by the senior author and described in detail by Troelsen et al. (2008).

**Figure 2.**
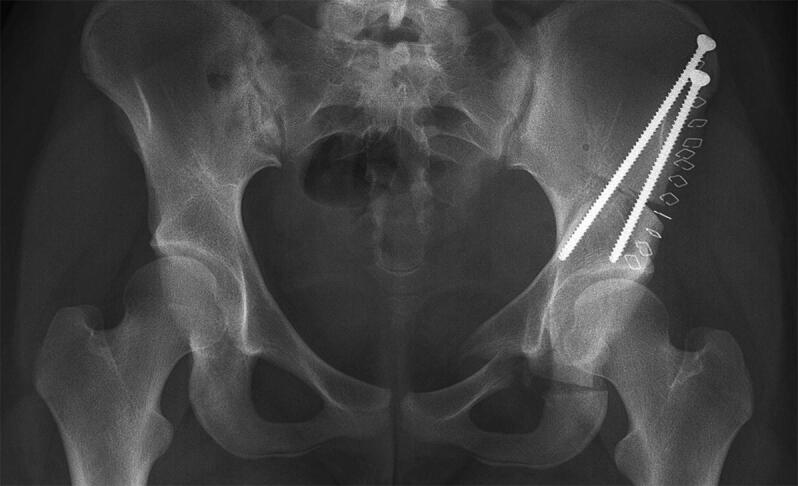
Minimally invasive periacetabular osteotomy showing osteotomy of pubis, ischium, and ilium and redirection of acetabulum and 2 screws for fixation.

Figure 2 illustrates a typical postoperative radiograph.

During hospitalization, the patients started a physiotherapist-supervised exercise program that continued after discharge supplemented by a home-based exercise program; the patients were allowed 30 kg weight-bearing for 6–8 weeks postoperatively and then full weight-bearing was allowed (Mechlenburg et al. 2018).

### Outcomes

Time to THA surgery was the primary outcome in the hip survival analysis. Follow-up started on the day of PAO and was considered a failure if the patient had undergone THA.

Complications after PAO were investigated based on ICD-10 codes and surgical procedures registered in the DNPR. Complications were categorized into serious medical conditions including DVT and pulmonary embolism (DI802, DI803(F), DI809, DI828, DI829, DT817D, DT817C, DI260, DI269) 1 month following PAO and complications requiring surgical intervention and additional surgery in the hip and pelvis (KNE, KNF) to end of study. Furthermore, the categories were categorized into relevant overall descriptions since not all codes are mutually exclusive.

The PRO in this study was HOOS, which was obtained preoperatively and at 6 months, 2, 5, and 10 years postoperatively. If HOOS pain score at 2 years was ≤ 50, it was considered a failure, similar to previously used WOMAC pain ≥ 10 (Hartig-Andreasen et al. 2012). Rate of responders with a difference ≥ 9 between preoperative score and follow-up score at 2 years was estimated from a minimally clinically important difference (MCID) of 9 on the HOOS subscale pain (Clohisy et al. 2017).

HOOS is a validated measurement for patients suffering from OA (Beyer et al. 2008). HOOS consists of 40 items and assesses 5 separate patient relevant dimensions: pain (10 items), symptoms (5 items), activities of daily living (ADL) (17 items), sport/recreation function (4 items), and hip-related quality of life (QoL) (4 items). Responses to items are given using a 5-point Likert scale (no, mild, moderate, severe, and extreme). The HOOS score on each subscale is a score from 0, indicating extreme problems, to 100 indicating no problems. Missing data are treated as such; provided at least 50% of the items are completed within a subscale, a mean score can be calculated and in the case of 2 answers, the smallest one was selected (indicating worse score).

### Statistics

Normally distributed data are presented as means (range), non-normally distributed data are presented as medians with interquartile ranges (IQR), and categorical data are presented as numbers with percentages. The cumulative hip survivorship was calculated using Kaplan–Meier survival analysis with THA as the endpoint. Cox’s proportional hazard regression analysis was used to compute hazard ratios and 95% confidence intervals (CI) for sex and age. The hips were analyzed as independent observations (Lie et al. 2004). Censorship was conducted at emigration, death, or end of study, whichever came first. The PAO was categorized as a failure if a patient reported a HOOS pain score ≤ 50. HOOS development over time was tested using univariate ANOVA. HOOS pain at 2 years was compared with preoperative HOOS using a paired t-test. At 2 years, the rate of responders was estimated using a MCID of 9 points.

All statistical analyses were performed using STATA/IC, version 15.1 (StataCorp LLC, College Station, TX, USA). For all risk estimates, a 95% CI was estimated. The level of significance was set at p < 0.05.

### Ethics, funding, and potential conflicts of interest

In an accordance with the General Data Protection Regulation in European countries, the Danish Data Protection Agency gave permission to handle the personal data (case no. 1-16-02-626-18). As the study was based on registry data, ethical approval is not needed according to Danish law. This study received no funding and the authors had no conflicts of interest.

## Results

### Demographics

Median patient age was 32 years (13–59) and the proportion of men was 15%. Demographic and preoperative data are presented in Table 1.

**Table 1.** Demographic and preoperative data concerning the 1,385 hips operated with periacetabular osteotomy. Values are n (%) unless otherwise specified

Covariates	Value
Age at time of surgery	
median (IQR)	32 (23–40)
range	(13–59
Sex	
female	1,175 (85)
male	210 (15)
Side of operation	
right	770 (56)
left	615 (44)
Additional covariates included after 2010	
Educational level (n = 617)	
1. General certificate of secondary education	101 (16)
2. Upper secondary school leaving	98 (16)
3. Vocational upper secondary education	91 (15)
4. Short-cycle higher education	80 (13)
5. Medium-cycle higher education	91 (15)
6. Bachelor education	92 (15)
7. Long-cycle higher education	60 (9)
8. PhD program	4 (1)
Body mass index (n = 617)	
mean (range)	23 (16–34)
Osteoarthritis score ^a^ (n = 592)	
Grade 0	571 (95)
Grade 1 or 2	21 (5)
VAS pain, median (IQR) (n = 618)	
pain at rest	35 (19–56)
pain during activity	75 (61–86)
Impingement test (n = 592)	
positive	562 (95)
negative	30 (5)
Previous treatment in the same hip (n = 592)	
yes	27 (5)
no	565 (95)
Center–edge angle (°) (n = 592)	
median (IQR)	19 (15–20)
range	(–10 to 25
Acetabular index angle (°) (n = 592)	
median (IQR)	15 (12–20)
range	(0–40

**^a^**Score according to Tönnis 1987

If data were not available for all 1,385 hips, n is stated.

IQR = interquartile range

VAS = visual analogue scale

### Hip survival

In the study period, 73 of the 1,385 hips were converted to THA. The mean time from PAO to THA surgery was 5 years (0.6–14) after PAO. The mean follow-up time was 5 years (0.03–14).

**Figure 3.**
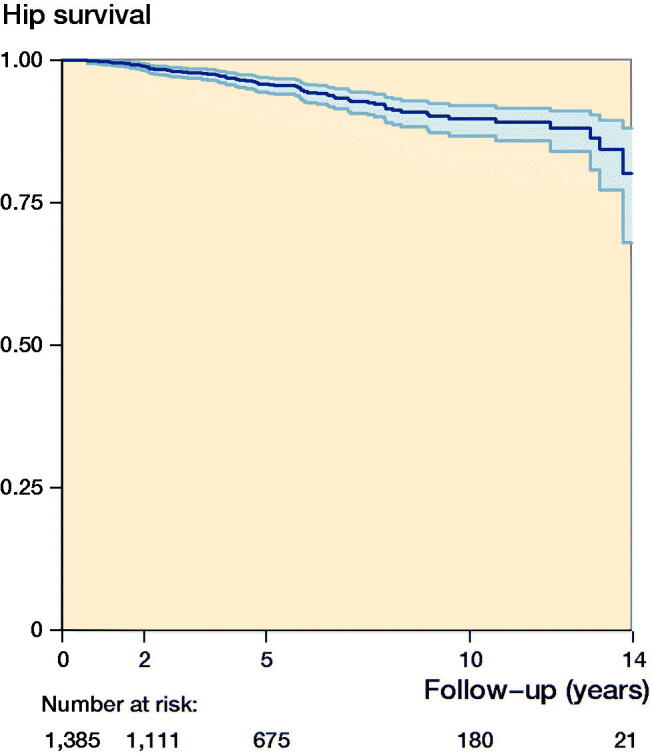
Kaplan–Meier survivorship curve (with CI) with conversion to total hip arthroplasty as endpoint for 1,385 hips after periacetabular osteotomy. Each decrease corresponds to a conversion to total hip arthroplasty. The number of hips at risk remaining for every year of follow-up is given below the x-axis. The hip survival rate is 80% (CI 68–88) at 14 years.

**Figure 4.**
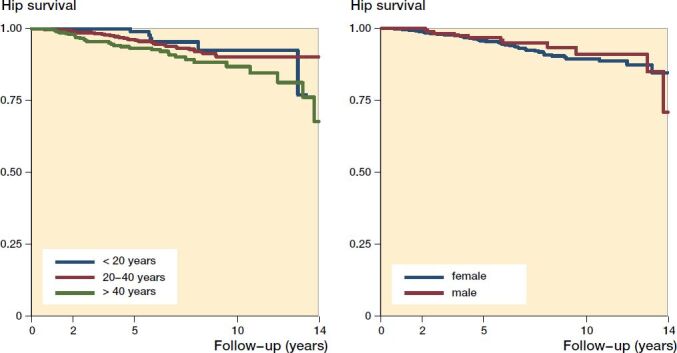
Kaplan–Meier survivorship curve, with conversion to total hip arthroplasty as endpoint for 1,385 hips after periacetabular osteotomy divided according to the age groups < 20 years, 20–40 years, > 40 years at surgery (left) and for each sex (right). Each decrease corresponds to a conversion to total hip arthroplasty. Log-rank test between age groups showed a p-value of 0.03 indicating a significant difference in survival and between the 2 sexes a p-value of 0.6 indicating no significant difference.

The Kaplan–Meier analysis with THA defined as failure showed a cumulative hip survival rate of 80% (CI 68–88) at 14 years for the entire cohort of 1,385 hips (Figure 3). The cumulative hip survival rate at 2-, 5- and 10-years’ follow-up was 99% (CI 98–99), 96% (CI 94–97), and 90% (CI 87–92), respectively. The hazard ratio for conversion to THA for women compared with men was 0.84 (CI 0.44–1.60). The hazard ratio for conversion to THA for the age group < 20 compared with the age groups 20–40 and > 40 was 1.4 (CI 0.6–3.4) and 2.5 (1.03–6.0), respectively (Figure 4).

### Complications and additional surgery after PAO

Complications and additional surgeries are listed in Table 2 and were found in 257 hips (243 patients) in the entire cohort. A medical condition (0.4%) consisting of DVT was observed in 6 hips (6 patients), but no pulmonary embolism was observed 1 month after PAO. The most common additional surgery was screw removal (13%) and hip arthroscopy (11%). Hip arthroscopy included resection of CAM and cartilage, partial synovectomy, and repair of labrum. The rare complications (1.1%) requiring surgical intervention are presented in Table 2. 4 hips (4 patients) had concomitant screw removal with revision PAO, open cheilectomy, treatment of non-union, and exploration of soft tissue, respectively. 25 hips (25 patients) had concomitant hip arthroscopy with screw removal. 60 hips (55 patients) had more than 1 complication or additional surgery. Of the 73 hips with conversion to THA, 45 hips (42 patients) had a complication or additional surgery prior to conversion to THA.

**Table 2.** Complications or additional surgery after periacetabular osteotomy (PAO) surgery on 1,385 hips

Type of complication	n (% of entire cohort)	Period min–max
Additional surgery		
Screw removal	173 (12.5)	0.3–11 years
Hip arthroscopy	154 (11.1)	0.4–9 years
Total hip arthroplasty	73 (5.3)	0.6–14 years
Open cheilectomy	1 (0.07)	6 years
Complications		
Nonunion	6 (0.4)	0.5–7 years
Superficial wound infection	3 (0.2)	23–89 days
Revision PAO	2 (0.1)	
< 1 month after PAO	1 (0.07)	3 days
> 1 month after PAO	1 (0.07)	0.7 years
Bleeding from corona mortis (coiled)	1 (0.07)	0 days
Open exploration of soft tissue	1 (0.07)	1 year
Unrelated to PAO surgery	11 (0.9)	
Z-plasty of the iliotibial band	8 (0.6)	0.8–10 years
Soft tissue biopsy	1 (0.07)	6 years
Femoral fracture	1 (0.07)	6 years
Tumor excision	1 (0.07)	7 years

#### Hip disability and Osteoarthritis Outcome Score

**Figure 5.**
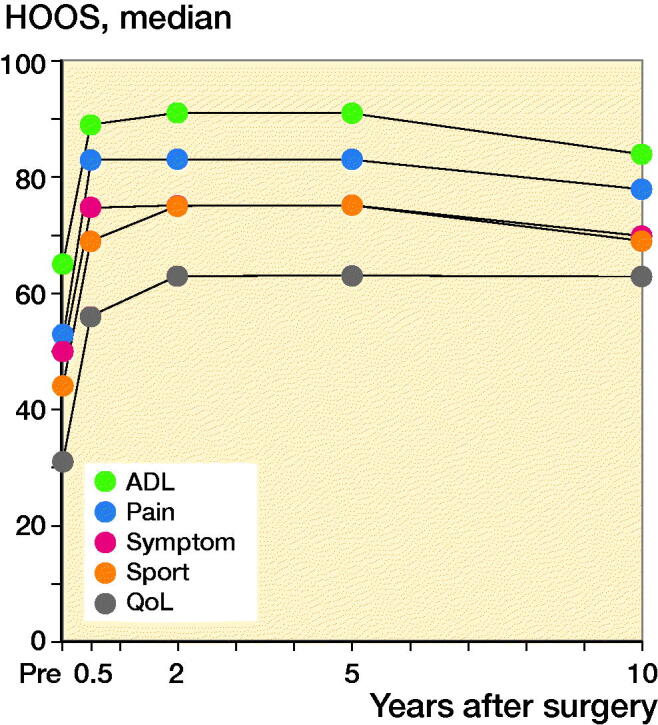
Graph showing the median scores preoperatively and at follow-up times for each Hip disability and Osteoarthritis Outcome Score subscale. Each Hip disability and Osteoarthritis Outcome Score showed a significant development over time for all subscales from preoperatively to 5 years with a p-value < 0.001.

The HOOS preoperatively and at follow-up is presented in Table 3. Each subscale shows that the preoperative score increased up to 6 months postoperatively and then plateaued from 6 months to 10 years (Figure 5). At 2 years’ follow-up (n = 624), 86% of the preserved hips scored no pain or low pain. From preoperatively to 2 years’ follow-up, the HOOS pain score (n = 427) improved significantly by 26 points (CI 24–28). At 2 years’ follow-up, 77% of the preserved hips reported an improvement of at least 9 points in HOOS pain compared with the preoperative score.

**Table 3.** Hip disability and Osteoarthritis Outcome Score (HOOS) preoperatively and at follow-up. Values are median (IQR)

			Follow-up time		
HOOS	Preop.	6 months	2 years	5 years	10 years
subscale	n = 599 (59%)	n = 656 (64%)	n = 643 (70%)	n = 528 (73%)	n = 197 (95%)
Pain	53 (40–65)	83 (68–93)	83 (65–95)	83 (63–95)	78 (63–93)
Symptoms	50 (35–65)	75 (60–85)	75 (55–90)	75 (55–90)	70 (50–85)
ADL	65 (47–78)	89 (76–97)	91 (74–97)	91 (71–97)	84 (66–96)
Sport/rec.	44 (25–56)	69 (50–88)	75 (50–94)	75 (50–94)	69 (44–88)
QoL	31 (19–44)	56 (38–75)	63 (38–81)	63 (44–81)	63 (44–81)

ADL = activities of daily living

QoL = hip-related quality of life

Completeness of HOOS is presented as n (% of possible answers).

## Discussion

The aim of this study was to describe the outcome after PAO surgery by estimating hip survival rate, complications, and additional hip surgery rates, as well as reporting hip function. At 14 years’ follow-up, we found a survival rate of 80% (CI 68–88%). The survival analysis showed a statistically significant difference in survival between age groups; thus, the risk of THA increases with age. In this cohort, complications such as DVT had a rate of 0.4% and the risk of revision PAO was 0.1%. The rates showed that the most common additional surgery was screw removal (13%) and additional hip arthroscopy (11%). HOOS clearly demonstrated that most patients experienced a significant improvement in HOOS pain score from preoperatively to follow-up at 2 years with a mean difference of 26 (CI 24–28). At 2 years’ follow-up, 86% of the preserved hips had no pain or a low pain score, defined by HOOS pain > 50.

The Kaplan–Meier analysis showed a survival rate of 80% (CI 68–88) at 14 years. This is slightly better than that reported by Steppacher et al. (2008), who found a 15-year survival rate of 77% and worse than that found by Wells et al. (2018a) of 92% at 15 years. At 10 years’ follow-up, our study found a survival rate of 90% (CI 87–92). This corresponds to results in previous studies ranging from 78% to 95% (Matheney et al. 2009, Hartig-Andreasen et al. 2012, Albers et al. 2013, Grammatopoulos et al. 2016, Ziran et al. 2018, Wells et al. 2018a). The difference in survival rates in our study compared with other studies could be because these studies included a small number of hips (between 68 and 401) (Table 4, see Supplementary data) compared with the 1,385 hips included in this study. The difference could also be explained by some studies being conducted on patients who were operated during the surgical learning curve, leading to an underestimation of the survival rate (Steppacher et al. 2008, Hartig-Andreasen et al. 2012, Albers et al. 2013, Grammatopoulos et al. 2016, Ziran et al. 2018). The high survival rate found by Wells et al. (2018a), could be explained by the fact that 13% (22 hips) were lost to follow-up, and these hips could potentially have had a THA, leading to an overestimation in the hip survival rate. We have 100% follow-up. Consistent with the literature, we found that increasing age was a predictor for failure of PAO with an HR of 2.5 (CI 1.0–6.0). This supports an upper age limit for patients undergoing PAO (Steppacher et al. 2008, Matheney et al. 2009, Hartig-Andreasen et al. 2012, Albers et al. 2013, Ziran et al. 2018). Moreover, our survival rate (80%) surpasses the 15-year implant survival rate after THA which was reported to be 64% in young patients below 35 treated for symptomatic hip dysplasia (Swarup et al. 2016).

Among our patients, 0.4% experienced DVT and no pulmonary embolism was experienced within 1 month after PAO. This corresponds well with results reported by Clohisy et al. (2017) where 0.5% experienced pulmonary embolism and 0.3% experienced DVT. Zaltz et al. (2014) found that 3 patients (1.5%) experienced DVT within the first 10 weeks after PAO surgery. The higher rate could be because indications for PAO other than symptomatic hip dysplasia were included. Clohisy et al. (2009) found that the most common moderate complication was the removal of symptomatic hardware. This is supported by our study, where screw removal was undertaken in 13% and was the most common additional surgery. 0.1% had a revision PAO; Wells et al. (2018b) found that 2% had revision PAO. 0.4% at our institution had a nonunion requiring reoperation. Wells et al. (2018b) found that 3% had a nonunion requiring open reduction. We do not perform concomitant arthroscopy at the time of PAO, and therefore found it relevant to investigate how many patients underwent hip arthroscopy after PAO. 11% of our patients underwent hip arthroscopy. Clohisy et al. (2017) found that 2% underwent hip arthroscopy due to persistent pain; however, 18% of the included patients had concomitant hip arthroscopy with their PAO surgery. Matheney et al. (2009) found that 11% underwent arthroscopy after PAO at a mean of 7 years, which is similar to the numbers found in the present study.

We found that 86% of the preserved hips had no pain or a low pain score. This rate corresponds well to previous results defined by a WOMAC score ≤ 10, with 84–88% preserved hips (Matheney et al. 2009, Hartig-Andreasen et al. 2012, Wells et al. 2018a) and a Merle d’Aubigné–Postel score < 15 ranging from 91–94% preserved hips (Albers et al. 2013, Lerch et al. 2017). The median HOOS score on all subscales showed a significant development over time. The mean change from preoperative to 2 years’ follow-up was 26 points (CI 24–28) for the HOOS pain score, which is in line with the 28 points change from the preoperative to the mean 2.6 years’ follow-up for HOOS pain reported by Clohisy et al. (2017).

### Strengths and limitations

The long-term follow-up of 14 years allowed us to assess complications of the PAO surgery and describe how many patients had additional hip surgery. The inclusion of PRO in this study is a strength, allowing for a secondary endpoint and for clinicians to make a more distinct conclusion regarding the success and failure of PAO. Furthermore, our study involved a large number of patients undergoing PAO. Merging our institutional database with the DNPR allowed complete follow-up on all patients and a high level of completeness (Schmidt et al. 2014, 2015).

Despite the high level of completeness, there is a risk of information bias. The DNPR uses SKS codes but there are potential differences that arise over time and among hospital departments, since codes are not mutually exclusive (Schmidt et al. 2015). Furthermore, a limitation in this study is that it includes only DVT, pulmonary embolism, additional surgery, and complications requiring surgical intervention in the hip. Another limitation concerns the data from the institutional database. Because the database was created in 2010, and patients operated before 2010 were first imputed in 2014, there might thus be a lack of completeness in the variables prospectively gathered after 2010. This limits the analysis on the HOOS data, where a small number of patients with 5-year follow-up had completed both the pre- and postoperative HOOS and none of the patients with 10-years’ follow-up had completed a preoperative HOOS.

## Summary

To our knowledge, this is the largest prospective follow-up study on outcomes after PAO surgery. In conclusion, PAO preserved 4 of 5 hips at 14-years’ follow-up. Furthermore, the minimally invasive PAO technique is safe (1.1% requiring reoperation) and was also shown to be effective with good clinical results. This study demonstrates that carefully selected patients will demonstrate good survivorship and that higher age leads to lower survivorship. Furthermore, the most common additional surgery was screw removal and hip arthroscopy. The majority of patients with preserved hips had no or low pain.

## Supplementary Material

Supplemental Material

## Supplementary data

Table 4 is available as supplementary data in the online version of this article, http://dx.doi.org/10.1080/17453674.2020.1731159

Table 4. Studies reporting survival rates after periacetabular osteotomy
