# Supplementary material for: 14-year hip survivorship after periacetabular osteotomy: a follow-up study on 1,385 hips
Source: Acta Orthop. 2020 Feb 28;91(3):299–305. doi: 10.1080/17453674.2020.1731159 (PMC8023930; doi:10.1080/17453674.2020.1731159)
Supplement: Supplemental Material [file IORT_A_1731159_SM3914.pdf]

Supplementary data

Table 4. Studies reporting survival rates after periacetabular osteotomy

Author (year)	Number of hips (patients)	THA	Lost to follow-up hips (%)	Age at surgery mean (range)	Follow-up mean (range/SD)	Survival rate (%)
Steppacher et al. (2008)	68 (58)	26	5 (7)	29 (13–56)	20 (19–23)	5 years: 93<br>10 years: 87<br>15 years: 77<br>20 years: 60
Matheney et al (2009)	135 (109)	33	23 (17)	27 (10–45)	9 (2.2)	5 years: 96<br>10 years: 84
Troelsen et al. (2009)	116 (96)	17	0 (0)	30 (14–57)	7 (5–9)	5 years: 91<br>9 years: 82
Hartig-Andreasen et al. (2012)	401 (316)	69	0 (0)	34 (13–61)	8 (4–12)	12 years: 75
Albers et al. (2013)	43 (42)	3	4 (2)	28 (13–44)	11 (10–14)	5 years: 95<br>10 years: 90
	122 (105)	16				5 years: 86<br>10 years: 78
Dahl et al. (2014)	122 (89)	11	5 (4)	31 (13–49)	7 (2.1)	12 years: 85
Beaulé et al. (2015)	72 (67)	1	2 (3)	32 (14–54)	5 (1–8.3)	5 years: 94<br>8 years: 86
Grammatopoulus et al. (2016)	68 (57)	4 [a]	2 (3)	25 (15–41)	8 (2–18)	10 years: 93
Lerch et al. (2017)	75 (63)	42	6 (8) [b]	29 (13–56)	29 (27–32)	30 years: 29
Ziran et al. (2018)	302 (258)	54	176 (41) [c]	33 (13–63)	11 (2–27)	10 years: 86<br>20 years: 60
Wells et al. (2018a)	154 (129)	8	22 (14)	26 (10–60)	10 (4–20)	5 years: 97<br>10 years: 95<br>15 years: 92
Isaksen et al. (2018)	69 (59)	9	3 (5) [c]	32 (14–44)	7.4 (2–15)	8 years: 84
Current study	1,385 (1,126)	73	0 (0)	32 (13–59)	5.4 (0.03–14)	5 years: 96<br>10 years: 90<br>14 years: 80

[a] THA and hip resurfacing.
[b] Questionnaire only.
[c] Calculated on number of patients
